# Custom-made acetabular implants for revision total hip arthroplasty: postoperative evaluation of the accuracy of implant positioning

**DOI:** 10.1186/s12891-025-09250-2

**Published:** 2025-10-13

**Authors:** Benjamin Schlossmacher, Igor Lazic, Christian Suren, Rainer Burgkart, Florian Pohlig, Rüdiger von Eisenhart-Rothe, Peter M. Prodinger

**Affiliations:** 1 Department of Orthopaedics and Sports Orthopaedics, Klinikum rechts der Isar, Technical University of Munich, School of Medicine, Ismaninger Str. 22, München, 81675 Germany; 2 Zentrum für Orthopädie & Sportmedizin (ZFOS), Munich, Germany; 3 Schön Klinik Harlaching, Munich, Germany; 4 Department of Orthopaedic Surgery, Krankenhaus Agatharied, Hausham, Germany

**Keywords:** Total hip arthroplasty, Custom implants, Acetabular bone defect, Revision, Positioning

## Abstract

**Background:**

The increasing need for revision total hip arthroplasty due to complications such as bone defects following implant loosening has – among others – led to the development of custom-made acetabular implants. This study evaluated the accuracy of implant positioning measured on AP radiographs and the respective clinical outcomes.

**Methods:**

A retrospective analysis of 31 cases involving severe acetabular bone loss (Paprosky type IIIa/ IIIb) was conducted. Preoperative planning was based on CT scans, and postoperative evaluation was performed via AP radiographs, focusing on Lewinnek’s safe zone for anteversion (AV) and inclination (INCL).

**Results:**

Results showed that 87.1% of the implants were positioned within or on the border of Lewinnek’s safe zone for AV and INCL. Mean AV (SD) was 13.9° (8.7°), mean INCL (SD) 46.9° (6.1°). Mean deviations between planning and postoperative results were 7.4° for AV (SD) (5.5°; p = 0.704) and 3.7° for INCL (SD) (5.2°; p = 0.068). Implant survival was 96.7% over a median follow-up (IQR) of 43.0 months (65.0).

**Conclusions:**

Correct positioning of customized acetabular implants could be achieved via assessment on plain AP radiographs using a superimposition method. The results were further emphasized by the low rate of mechanical complications and high implant survival. Lewinnek’s safe zone is a good guideline for proper cup positioning but should not be prioritized over primary stability of the implant. Accurate planning is key to achieving both a satisfying position and sufficient osseous integration. Further research involving higher case numbers through a multi-center approach is needed to draw definitive conclusions.

## Introduction

Due to the continual rise in life expectancy and the subsequent increase in clinically evident osteoarthritis of the hip, the global number of arthroplasties has consistently increased in recent years [1–3].

A significant proportion of these reconstructions yield excellent results [4, 5]. However, despite all the progress made in treatment, there is a relevant complication rate, which often results in the need for partial or complete replacement of the implant [6, 7].

Extensive osseous defects often occur during implant removal, following wear disease or even in primary situations such as chronic high hip dislocations, which can be treated by various procedures [8–10]. While many surgeons prefer to augment the defect with autologous or allogenic bone material and a conventional shell, others are using modular cups with augments to address the distinct acetabular defect. Those augments can be either conventional stock models or – as presented in a recent study with promising results – custom made to the distinct defect [11].

In some cases, acetabular bone loss exceeds the possibilities of such conventional augmentation methods.

In those cases, a custom-made implant with an integrated, macroporous augment as a monolithic design fitted to the defect zone and additional flanges and pegs has become a valid option in recent years [11, 12].

With increasing technical possibilities of three-dimensional imaging and complex image processing, the obstacles to offering such a solution tend to decrease.

Intraoperative positioning of those implants presents one of the major challenges: the implant should fit the preoperatively measured defect volume. However, bone quality, the impaired status of the surrounding soft tissue and seemingly restricted intraoperative imaging solutions can substantially complicate intraoperative positioning.

The aim of correct cup positioning must be a sufficiently restored centre of rotation (COR) as well as a functionally correct anteversion (AV) and inclination (INCL) of the cup to avoid complications such as hip dislocation or limping due to muscular imbalances. Thus, in revision arthroplasties with extensive bone loss, osseous landmarks are often missing and therefore further complicate the positioning of the cup.

Few studies have reported on the correct positioning of custom-made implants and have mostly used pre- and postoperative CT scans to assess the actual position [13–22]. However, CT scans continue to involve disproportionate radiation exposure in the hip area, which prevents routine use in follow-up care. Comparisons between plain radiographs and CT scans for postoperative evaluation are scarce. Only one available study on custom-implants has shown acceptably small deviations for both modalities with the same methodology as ours [13]. Additionally, outcomes such as rerevision rates in those cohorts have been infrequently reported.

Therefore, our aim was to evaluate the accuracy of implant positioning using only AP radiographs of the pelvis as this remains the most accessible imaging option in daily clinical practice and to relate those results with postoperative outcomes. Expected benefits were the reduced use of postoperative CT scans and a broader use of custom-made implants as the fear of malpositioning decreases.

## Materials and methods

A total of 45 consecutive acetabular revisions at our institution between 2013 and 2018 were analysed retrospectively.

According to the classification by Paprosky et al. [23], all defects were classified as type IIIa or IIIb. Aseptic and septic cases were included. In revision cases, indications for custom-made implants were large acetabular bone defects following septic or aseptic loosening of the THA. Primary indications were massive bone defects in osteoarthritis and postoperative defects following tumour resection.

Among those patients, 14 were excluded because of limited imaging data. Those included technical issues with the matching of anonymised images (*n* = 4), significant malpositioning of the pelvis due to patients not being able to follow standardized positioning routine (*n* = 6) and extensive tumour resections without the use of a classical COR as comparison (*n* = 4).

The study was approved by the local institution’s Ethics Committee (reference no. 714/20 S) and was conducted in accordance with the Helsinki Declaration.

Preoperative CT scans were performed to illustrate the exact dimensions of the defect zone in all cases. Preprocessing of the imaging data, planning, and manufacturing of the customized implant were performed by AQ Solutions, Huerth, Germany. Upon agreement on surgical requirements and technical feasibility, the customized implants were produced. This included the placement and orientation of screws and the iliac peg, as well as regions for porous metal augmentation. The implant was designed as a monobloc component made of titanium alloy to be manufactured using a selective laser melting process.

**Fig. 1:**
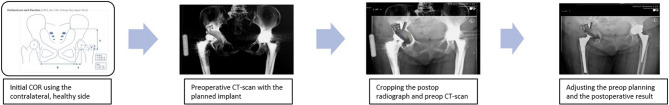
Process of the initial calculation of the centre of rotation, positioning of the planned implant and comparison to the final result

After surgery, partial weight-bearing was ordered for six weeks. Regular postoperative radiographs were taken 3 days, 6 weeks and one year after surgery. The initial COR for positioning of the custom implant was calculated following the method of Pierchon et al. [24] (Fig. 1), AV was measured according to Liaw et al. [25] (mathematical ellipse approach) and INCL was measured according to Lewinnek et al. [26] (angle between the horizontal reference line and line across rim of the cup) using AP radiographs of the pelvis taken 6 weeks postoperatively. Patient positioning was standardized following our in-house routine (supine position, source-to-image-distance 1 m, no angulation). The results were compared to the predefined measures of the preoperative CT scan by superimposition of the postoperative radiograph using the manufacturer’s in-house planning software (Fig. 1). Because of the variability in patient posture and the intricate shapes of custom implants, AQ Implants utilized a specialized software to evaluate implant positioning. The software not only allowed for the creation of 3D bone models from CT scans – essential for planning custom implants with respect to each patient’s physiological center of rotation and any leg length discrepancies – but also enabled the integration of 2D X-rays with superimposed 3D CT data. The workflow began by importing and scaling the 2D X-rays based on the specific implant measurements. Next, 3D reconstructions of the pelvic bone from post-operative CT scans, including the positioned implant, were added and aligned to match the pelvic orientation seen on the X-ray images. During this alignment, care was taken to ensure that anatomical landmarks matched up between the 2D X-rays and the 3D pelvic model, using an outline projection for accuracy, with the implant model kept in its planned position.

Subsequently, the pelvic orientation was fixed, and only the implant’s orientation was fine-tuned to correspond to its actual placement as observed in the X-rays. While exact anteroposterior alignment of radiographs is challenging, the distinctive shape of the implant, as well as features such as metal augmentation and screw fixation, makes an isolated shift in the AP direction unlikely without altering other parameters. The resulting changes – both translational and rotational – were then recorded and used to determine any differences in the center of rotation, as well as changes in the anteversion and inclination angles of the implant cup. All computed measurements were referenced back to the original CT scan data, and any arbitrary tilting of the pelvis present in the X-rays was accounted for by relying on the transformed CT coordinate system.

Lewinnek’s safe zone for AV and INCL was used as a reference for correct cup positioning [26]. In the original paper, ranges of 5–25° and 30–50° were proposed for AV and INCL, respectively. We considered our results to either be within, on the confines or outside the safe zone. We analysed the cup survival and revision rates but no clinical outcome scores were reported due to restricted data availability. All implants were preoperatively planned to be inside the aforementioned safe zone.

A Shapiro-Wilk-test was performed for the testing on normally distributed variables. If so, the mean and standard deviation (SD) were given, else the median and interquartile range (IQR).

The Mann-Whitney-U test and t-test were performed for all continuous variables. Values of α < 0.05 were considered to indicate statistical significance. The statistical analysis was carried out using IBM SPSS Statistics for Windows, version 27.0 (Armonk, New York: IBM Corporation).

## Results

31 cases were finally included in this study. The mean age (SD) at surgery was 66.2 (10.3) years. Patient demographics are demonstrated in Table 1. The median (IQR) follow-up was 43.0 (0–109) months. The mean time (SD) between the indication for implantation of a custom-made acetabular implant and final surgical implantation was 52.1 (29.7) days. The mean (SD) number of prior surgeries was 3.0 (2).

**Table 1:** Patient demographics

Age in years (mean; SD)		66.2 (10.1)
ASA-score^1^ (*n*; %)		
	ASA I	0
	ASA II	20 (64.5%)
	ASA III	11 (35.5%)
	ASA IV & V	0
BMI^2^ in kg/m² (mean; SD)		26.6 (3.3)
Mean (SD) number of prior surgeries		3.0 (2.0)

^1^*ASA * American association of anesthesiologists, ^2^*BMI * Body mass index

The indications for acetabular revision were excessive bone loss following prosthetic joint infection (PJI) in 11 cases, aseptic loosening in 18 cases and tumour resection of the pelvis in two cases. One oncologic case was a solitary, fracture-prone breast-cancer metastasis, the other one a chondrosarcoma of the pelvis (G2).

For the surgical procedure, an anterolateral approach was used in 19 cases. 11 cases were operated on with a lateral approach, and in one case, a posterior approach was used.

In all cases a dual mobility cup was implanted into the acetabular component to prevent dislocation due to the extensive damage to the surrounding soft tissues.

Nine revisions had to be performed during the observed period. Overall, the revision rate was 29.0%. Implant survival was 96.7% after a median follow-up of 43 months.

In six cases, reinfections were revised without implant removal. In one case, recurrent hip joint dislocations necessitated an exchange of the mobile parts of the prosthesis. One revision was due to periprosthetic fracture of the femur without addressing the acetabular component. Last, one case had an aseptic loosening of the acetabular component after 8 years and was subsequently revised with the exchange of the implant.

Both cases of joint dislocation and aseptic loosening requiring re-revision were on the confines of Lewinnek’s safe zone (1: AV 5/INCL 50; 2: AV 25/INCL 50).

The mean (SD) preoperatively planned and postoperative AV of the customized implants was 14.5° (5.3°) and 13.9° (8.7°), respectively. With respect to the safe zone proposed by Lewinnek et al., 27 arthroplasties (87.1%) were within or on the confines of the safe zone, and four (12.9%) were outside of the proposed range. The mean difference between the planning and postoperative results regarding the AV (SD) was 7.4° (5.5°; *p* = 0.704).

The mean (SD) preoperatively planned and postoperative INCL was 44.8° (0.9°) and 46.9° (6.1°), respectively. 27 cases (87.1%) were within, and four (12.9%) were outside of the proposed range. The mean difference between planning and postoperative results regarding INCL (SD) was 3.7° (5.2°; *p* = 0.068), with a maximum deviation of 25° in one case.

No statistically significant differences were observed for pre- and postoperative AV and INCL.

With respect to the combined AV and INCL, 8 implants (25.8%) were not within Lewinnek’s safe zone with 4 each.

The mean (SD) preoperatively planned and postoperative leg length difference was − 2.1 (9.8) mm and − 5.3 (10.2) mm, respectively. The mean (SD) difference of the radiologic leg length between the preoperative planning and postoperative results was 4.5 (4.1) mm, with a maximum of 17.5 mm (*p* = 0.002).

The mean (SD) lateral and cranial deviations of the COR between the planned and postoperative results were 4.4 (3.3) mm and 4.5 (4.1) mm, respectively. Both differences were statistically significant (*p* < 0.001).

**Fig. 2:**
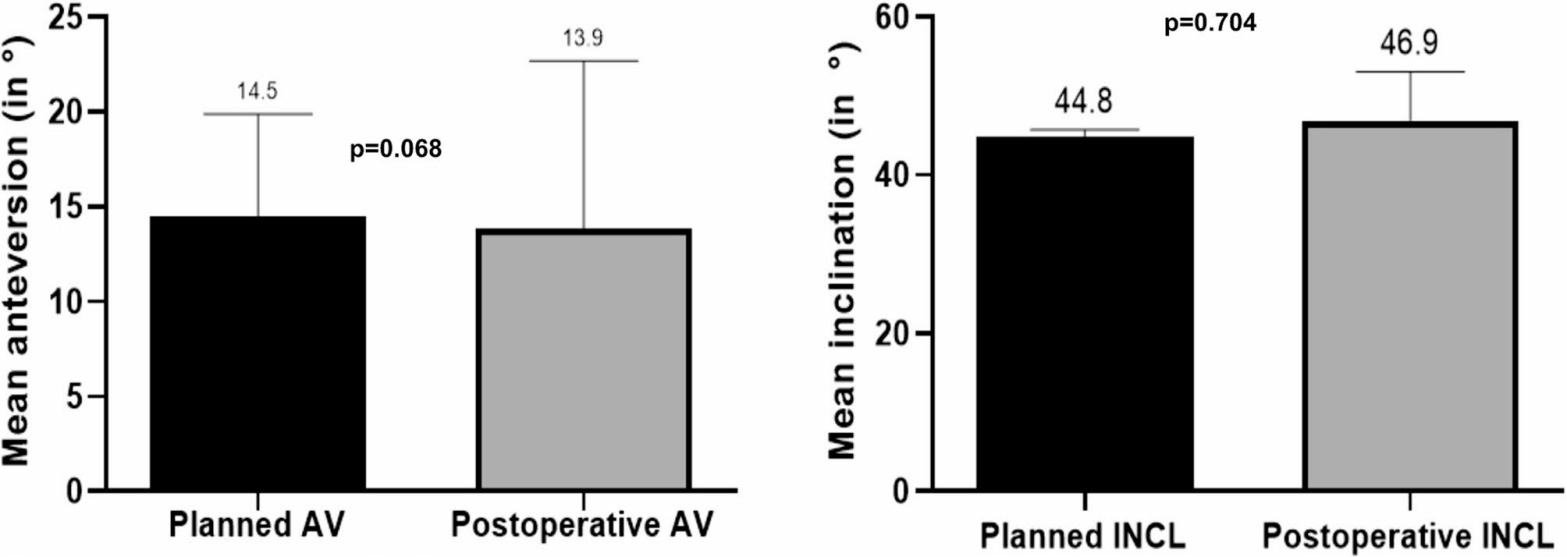
Mean inclination and anteversion for planned and postoperative results

**Fig. 3:**
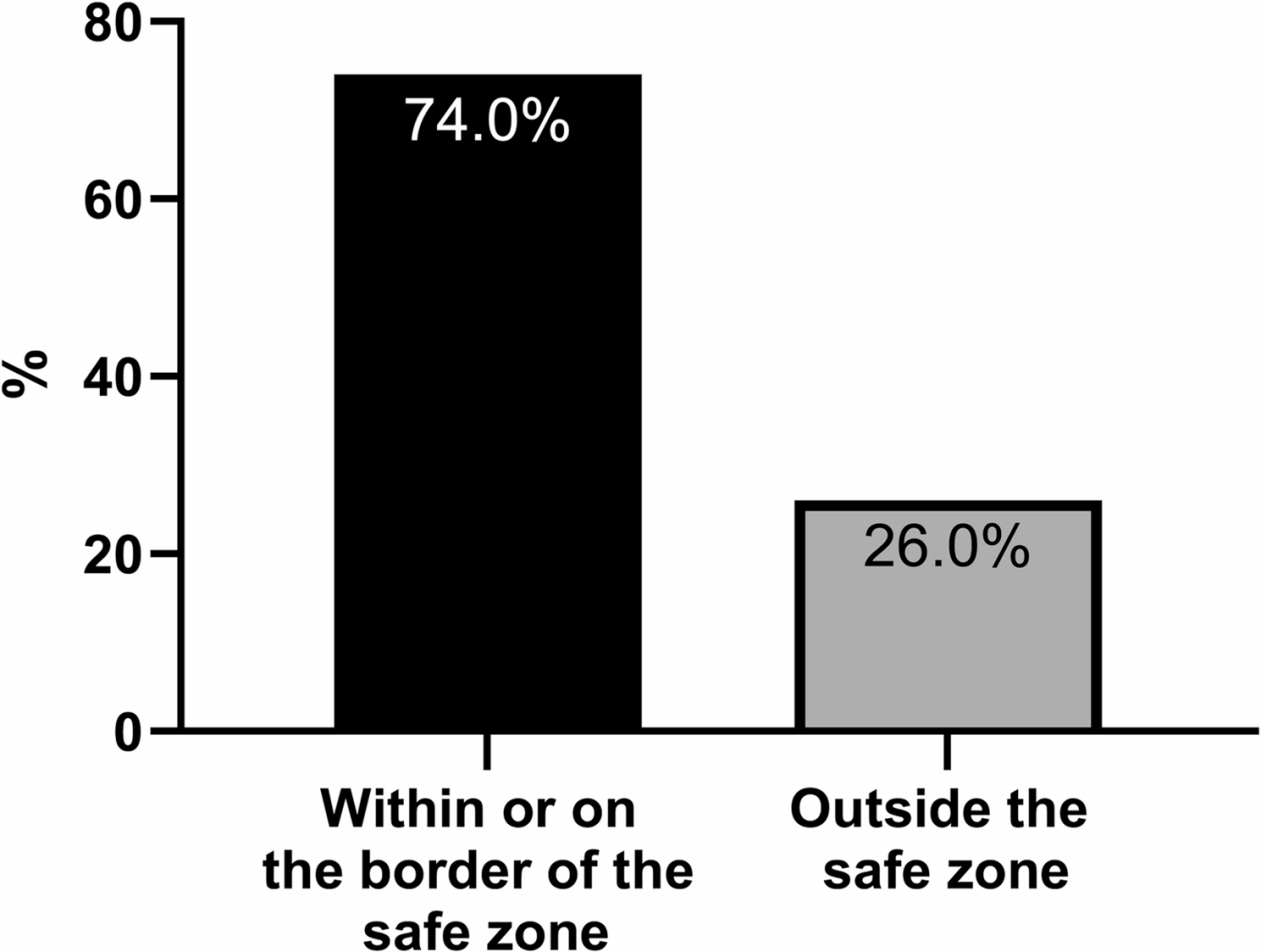
Positioning of the implants regarding Lewinnek’s safe zone (AV and INCL included)

The results are summarized in Table 2; Figs. 2 and 3.

**Table 2:** Results

Inclination		*p*-value
Planned mean (SD) in °	44.8 (0.9)	-
Postoperative mean (SD) in °	46.9 (6.0)	-
Range in °	35–70	-
Mean (SD) difference between planning and result in °	7.4 (5.5)	0.704
Inside Lewinnek’s safe zone (n; %)	27 (87.1%)	-
Confine of safe zone (n; %)	6 (19.4%)	-
Outside the safe zone (n; %)	4 (12.9%)	-
Anteversion		
Planned mean (SD) in °	14.5 (5.3)	-
Postoperative mean (SD) in °	13.9 (8.7)	-
Range in °	0–40	-
Mean (SD) difference between planning and result in °	3.7 (5.2)	0.068
Inside Lewinnek’s safe zone (n; %)	27 (87.1%)	-
Confine of safe zone (n; %)	9 (29.0%)	-
Outside the safe zone (n; %)	4 (12.9%)	-
Leg length		
Mean (range; SD) leg length discrepancy in mm	−5.3 (−31-13); (10.1)	**-**
Mean (SD) difference between planning and result in mm	4.5 (4.1)	**0.002**
COR^1^		
Mean (SD) lateral deviation between planning and result in mm	4.4 (3.4)	**< 0.001**
Mean (SD) cranial deviation between planning and result in mm	4.5 (4.2)	**< 0.001**
Revisions		
Any revision (n; %)	9 (29.0%)	-
Implant removal (n; %)	1 (3.2%)	-

^1^*COR * Centre of rotation

## Discussion

The proper positioning of individual acetabular components in revision THA is complex. The first finding of this study is that the positioning of these implants was generally successful, with no significant deviations from the preoperative planning, as validated by plain radiographs. This emphasizes the consistency of the surgical procedure in achieving proper implant positioning, even in cases involving complex anatomical considerations and large implants. In this context, plain radiographs seem to offer sufficient accuracy in the evaluation of the implant position, thus high radiation doses of CT scans might be avoided. CT scans are not feasible for each patient intra- and postoperatively for the sole purpose of controlling the implant position.

Further evidence for a valid postoperative implant assessment is the low implant revision rate. Only one implant had to be revised after eight years for aseptic loosening, accounting for an overall implant survival of 96.7%. The relatively high revision rate of almost 30% might derive from the inclusion of PJI cases as 6 out of 9 revisions were due to infection recurrence.

Overall, the reported revision rates in literature are lower. However, this is partly attributable to short follow-up periods reported compared to our study. Baauw et al. and Zampelis et al. did not report any re-revision of the custom-made implant without giving an exact follow-up, and Weber et al. reported no failure of the cup within the first six months after reimplantation [13–15]. Choi et al. found that 1 out of 24 cups (4.2%) had to be revised due to aseptic loosening following malpositioning (INCL 11°, AV 33°) after a follow-up of 7.6 years [16].

De Martino et al. performed a systematic review and reported a revision rate of 3.1% for aseptic loosening of custom-made acetabular cups with at least 12 months of follow-up. The overall revision rate was 17.3%, which is lower than that reported in our study [20]. Another possible explanation is the high rate of initial periprosthetic joint infections (11 out of 31 cases) as an indication for the implantation of a custom-made implant, which in turn significantly increases the risk for re-revision due to infection. The rate of mechanical complications on the other hand remained low with only one dislocation and one aseptic loosening after 8 years.

In a recent systematic review by Weintraub et al., 93 out of 592 custom-made acetabular implants had to be revised (15.7%) [27]. Once again, the lower rate of septic loosening as the indication for a custom implant (39/592; 6.6%) might present a valid explanation for the difference between this cohort and ours.

Notably, the majority of implants were positioned within Lewinnek’s safe zone. Nevertheless, approximately 26% of our cases could not be considered inside Lewinnek’s safe zone, with one case showing a final INCL of 70° and a radiographic leg length discrepancy of 17.5 mm. In this patient, a long interval between the planning of the acetabular component and the final implantation with several spacer exchange procedures caused a new osseous defect to develop. Therefore, positioning the cup as intended was not possible and a steep INCL had to be tolerated to provide sufficient stability and ingrowth of the implant.

For the other 7 cases outside the safe zone, there were 3 cases of severe soft tissue contractions and 1 oncologic case further complicating proper implant positioning. For the other 3, no such explanations for malpositioning could be identified. Especially such major discrepancies between planning and postoperative results show the risks of custom implants as they do not offer the possibility to react to unforeseen changes such as increasing bone loss.

While non-significant deviations from the originally planned position were seen, all but one case with major deviations of over 10° did not result in clinical and especially mechanical implant failure. While the case of posterior joint dislocation was still on the confines of Lewinnek’s safe zone (AV 5°/INCL 50°), the low AV might present an explanation for the posterior instability.

The results observed in this study are consistent with those of the literature on custom-made acetabular implants available to date. In a study by Baauw et al., 13 out of 16 patients were within Lewinnek’s safe zone, whereas three other studies reported 80%, 70%, and 56% success rates, respectively [13–16].

Despite the broad reference to Lewinnek’s safe zone in our study and a variety of other publications on implant positioning in primary and revision THA, recent articles have questioned the importance of the safe zone as a predictor of mechanical complications [28–30].

Several studies reporting implant positions within Lewinnek’s safe zone have shown divergent success rates for primary THA between 25% and 88% but not necessarily higher dislocation rates [31–33]. On the one hand, implants may be positioned outside the safe zone and remain stable; on the other hand, others are correctly positioned and dislocate, nevertheless [34].

Therefore, several other safe zones have been proposed with a focus on functional and patient-specific factors such as pelvic immobility following lumbar surgery [35, 36].

Unfortunately, studies evaluating safe zones for revision THA are scarce. Those published recommend using Lewinnek’s safe zone as an orientation but prioritizing functional aspects of the acetabulum in complex cases with patients having undergone several prior surgeries [34, 37].

Another frequently discussed variable is the leg length discrepancy. We have refrained from proposing a safe zone for maximum difference of leg length with regard to a risk for instability as the only dislocation in our cohort showed a difference of 1 mm and therefore, no valid conclusion can be drawn. Underlining the limited influence of the leg length discrepancy, a recent publication by Kaji et al. in a cohort of 12.500 THA has shown no significant influence of leg length or offset discrepancies on the risk for joint dislocation [38].

Additionally, custom-made acetabular implants such as those used at our hospital are combined with a cemented dual mobility cup. Therefore, final AV and INCL can be adjusted to a certain degree even for implants that cannot be positioned as intended. Previous studies have shown that the use of dual mobility cups significantly decreases the risk of hip joint dislocation in revision THA where major defects to the soft tissues are present [39].

While this paper focuses solely on custom-made acetabular implants, mostly consisting of a monobloc design with additional screws for fixation, other options present similarly promising results. For instance, other approaches – such as modular implants with a variety of augments or bone impaction grafting [11]. These alternatives can address large bone defects without resorting to costly custom implants and allow intraoperative flexibility by adding larger augments or additional allografts/autografts as needed, especially if the defect is larger than expected.

The positioning of the individual implants is often difficult because of insufficient preparation of the bone bed in the former acetabulum. Missing osseous landmarks further complicate the positioning as intended by the manufacturer.

Especially when the acetabular defects have been present for a longer period, soft tissue contractions are causing problems getting the implant into the correct position and sometimes necessitate soft tissue release that further destabilizes the joint. Soft tissue concerns are particularly problematic with individual implants as they are not put into consideration in the osseous planning process.

Further factors that prevent widespread use are the associated costs and the logistics that derive from ordering a custom implant. Cost coverage must be secured beforehand and is particularly dependent on the local health care system. In many cases, neither the insurance nor the patient can bear the cost. The acquisition of all images required planning and the manufacturing process of either 3D printing, or the above-mentioned laser melting process take significant time. As previously described, this might increase the risk for further bone loss and deterioration of the patient’s health status.

Another current topic in research is the robotic assisted THA. It should – in theory – provide improved and more reliable implant positioning. Nevertheless, further research on this emerging technique is necessary and the way towards robotic assisted revision THA remains long.

To conclude, meticulous planning of the implant via three-dimensional reconstructions as well as an evaluation of the desired functional cup position are the keys to successful surgery.

Ultimately, the surgeon must find a compromise between primary implant stability and cup positioning. A safe zone can be helpful here but should not be over-prioritized. Minor deviations did not seem to have any influence on the clinical outcome, especially regarding joint dislocations.

Regarding unforeseen changes between the planning and final surgery, even custom implants should offer the possibility to add augments for addressing additional bone loss. Manufacturers might put this into consideration when designing new implant designs.

### Limitations

Several limitations of this study should be acknowledged. First, the study’s retrospective design inherently carries limitations, including potential biases and data collection challenges without control over confounding variables. We included a consecutive series of patients to avoid additional bias. Second, the small cohort size limits the generalizability of the findings to a broader patient population. However, it is worth noting that this study benefits from a representative pool of patients, with 31 cases included, which is a relatively high number compared with the limited available literature to date.

Third, the absence of functional outcome scores and the short follow-up duration restrict our ability to draw conclusions about long-term clinical outcomes. Especially considering the influence of cup positioning on the range of motion as well as potential impingement, this should be an important factor in future studies and could provide further insights into this topic.

Fourth, postoperative CT scans were not sufficiently available as an additional group for controlling implant positioning. A minor discrepancy between postoperative CT scans and postoperative radiographs has already been demonstrated [13]. In this study, however, we analysed direct comparisons between preoperative planning images and postoperative radiographs in a significantly larger population.

The analysis on the manufacturer’s in-house software carries further limitations as it is not open to the public for further validation.

## Conclusion

In conclusion, our study shows promising results for the correct positioning of custom-made acetabular implants in revision THA when assessed via plain AP radiographs. We found a high implant survival rate of 96.7% further emphasizing the low rate of mechanical complications. While a recommendation on plain AP radiographs as routine follow-up can be given, CT scans should remain gold standard when exact measurements are necessary.

Lewinnek’s safe zone is a good guideline for proper cup positioning but should not be prioritized over the primary stability of the implant. Accurate planning is key to achieving both a satisfying position and sufficient osseous integration. The time between planning and final surgery should be kept as short as possible to avoid further, unforeseen bone loss.

Future research should further examine functional outcomes to gain insights into potential complications through insufficient implant positioning that might not cause surgical revision. Additionally, bigger cohorts through multi-center approaches are needed to improve generalizability in this particular field.

## Data Availability

The datasets used and/or analysed during the current study are available from the corresponding author on reasonable request.

## Abbreviations

AP: Anterior-posterior
ASA: American association of anaesthesiologists
AV: Anteversion
BMI: Body mass index
COR: Rentre of rotation
INCL: Inclination
IQR: Interquartile range
PJI: Periprosthetic joint infection
SD: Standard deviation
THA: Total hip arthroplasty
